# Mass Spectrometry-Based Untargeted Metabolomics and α-Glucosidase Inhibitory Activity of Lingzhi (*Ganoderma lingzhi*) During the Developmental Stages

**DOI:** 10.3390/molecules24112044

**Published:** 2019-05-29

**Authors:** Dedi Satria, Sonam Tamrakar, Hiroto Suhara, Shuhei Kaneko, Kuniyoshi Shimizu

**Affiliations:** 1 Division of Systematic Forest and Forest Products Sciences, Department of Agro-Environmental Sciences, Graduate School of Bioresource and Bioenvironmental Sciences, Kyushu University, Fukuoka 812-8581, Japan; dedi.satria@umsb.ac.id or dedi.satoria@gmail.com (D.S.); tamrakar.snm@gmail.com (S.T.); 2 Faculty of Health and Sciences, Muhammadiyah University of Sumatera Barat, Bukittinggi 26181, Indonesia; 3 Miyazaki Prefectural Wood Utilization Research Center, Miyazaki 885-0037, Japan; suhara-hiroto@pref.miyazaki.lg.jp; 4 Fukuoka Prefecture Forest Research & Extension Center, Fukuoka 818-8549, Japan; shu-k@kir.biglobe.ne.jp

**Keywords:** *Ganoderma lingzhi*, developmental stages, untargeted metabolomics, GC/MS, LC/IT-TOF-MS, α-glucosidase inhibitory activity

## Abstract

Lingzhi is a *Ganoderma* mushroom species which has a wide range of bioactivities. Analysis of the changes in metabolites during the developmental stages of lingzhi is important to understand the underlying mechanism of its biosynthesis, as well as its bioactivity. It may also provide valuable information for the cultivation efficiency of lingzhi. In this study, mass spectrometry based untargeted metabolomics was carried out to analyze the alteration of metabolites during developmental stages of lingzhi. Eight developmental stages were categorized on the basis of morphological changes; starting from mycelium stage to post-mature stage. GC/MS and LC/MS analyses along with multivariate analysis of lingzhi developmental stages were performed. Amino acids, organic acids, sugars, polyols, fatty acids, fatty alcohols, and some small polar metabolites were extracted as marker metabolites from GC/MS analysis, while, lanostane-type triterpenoids were observed in LC/MS analysis of lingzhi. The marker metabolites from untargeted analysis of lingzhi developmental stages were correlated with the α-glucosidase inhibitory activity. Two metabolites, compounds **34** and **35**, were identified as potential contributors of the α-glucosidase inhibitory activity. The current result shows that some metabolites are involved in the developmental process and α-glucosidase inhibitory activity of lingzhi.

## 1. Introduction

Lingzhi (*Ganoderma lingzhi*) is a non-edible woody mushroom native to the eastern parts of Asia, including China, Japan, and Korea [1,2]. For over two millennia, *G. lingzhi* has been traditionally used for medicinal purposes [3]. Recent studies of *G. lingzhi* show that it has a wide range of bioactivities such as anti-androgenic, anti-cancer, anti-hypertension, anti-virus, anti-melanocyte, and anti-diabetes [4,5,6]. In particular, *G. lingzhi* triterpenoids are known to be responsible for the inhibition of α-glucosidase and aldose reductase, leading to their anti-diabetic properties [7,8,9,10].

Owing to the wide range of bioactivities, the commercialization of *G. lingzhi* products is increasing rapidly [11]. Commercial products derived from mycelia, spores or fruiting bodies of *G. lingzhi* are mostly sourced from artificial cultivation [12]. The artificial cultivation not only alleviates the difficulties in obtaining wild *G. lingzhi* in nature, but also helps to fulfill the ever increasing global demand [12]. However, it is important to note that the quality of *G. lingzhi* products varies considerably depending on different strains, cultivation conditions, seasonal variations, as well as the variations in the developmental stage of the harvested fruiting bodies [13]. 

Metabolomics approaches by means of mass spectrometry (MS) have been gaining interest not only for analysis of plants, but also for mushrooms [14,15]. Gas chromatography-mass spectrometry (GC/MS) is a platform which is frequently used in metabolomic studies for the analysis of primary metabolites, whereas liquid chromatography-mass spectrometry (LC/MS) is commonly used for the analysis of secondary metabolites [16]. To increase the coverage of metabolites, the integration of GC/MS and LC/MS platforms has been reported frequently [17,18]. For instance, it has been used to analyze the alteration of metabolites during developmental stages of plants or ripening phases of fruit [19,20]. This analysis is important to understand the underlying mechanism of the biosynthesis of metabolites, as well as their bioactivity. 

Although, targeted analysis of some *G. lingzhi* triterpenoids during its various developmental stages has been reported before [13,21]; untargeted metabolomics studies analyzing the chemical changes of *G. lingzhi* at different developmental stages has not been reported to date. In this study, untargeted metabolomics was performed by using GC/MS and LC/IT-TOF-MS in combination with multivariate analysis, to investigate the changes of metabolites in *G. lingzhi* at different developmental stages. Moreover, we demonstrated the variability in alpha-glucosidase inhibitory activity throughout the developmental stages of *G. lingzhi*. 

## 2. Results

### 2.1. Primary Metabolite Profiling of G. lingzhi at the Eight Developmental Stages by GC/MS

**Figure 1:**
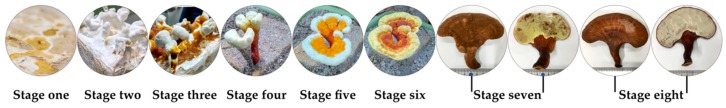
Experimental design of lingzhi (*Ganoderma lingzhi*) analysis harvested at eight developmental stages.

The morphological changes of the fruiting bodies of *G. lingzhi* during its development were categorized into eight stages, as shown in Figure 1: stage one (mycelia), stage two (primordia), stage three (bud-breaking, brown stipe), stage four (early cap formation), stage five (cap formation, white edge), stage six (immature stage, yellow edge), stage seven (mature stage, spore not dispersed), and stage eight (post-mature stage, after spore dispersed). 

**Figure 2:**
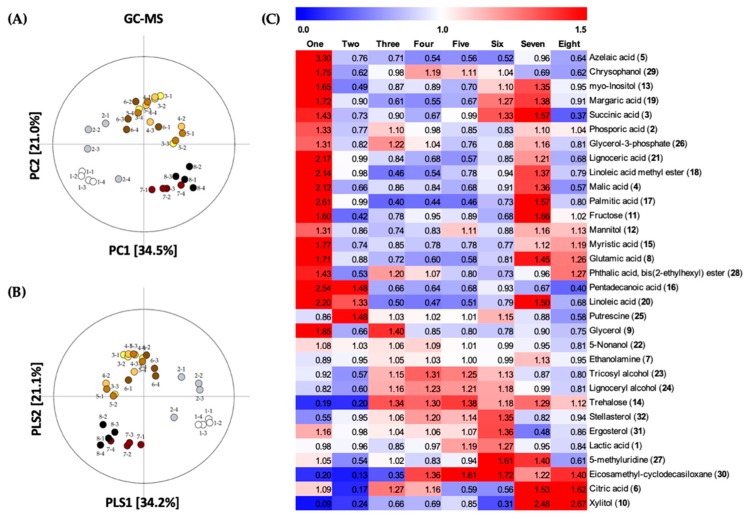
(**A**) PCA score plot and (**B**) PLS-DA score plot of each *G. lingzhi* samples at the eight developmental stages analyzed by GC/MS; (**C**) Heat maps of significantly different metabolites at the eight developmental stages of *G. lingzhi* from GC/MS analysis. Each column represents the developmental stage, and the fold change of average peak area denoted by the number and color of heat scale.

To investigate the changes of primary metabolites in *G. lingzhi* fruiting bodies at the eight developmental stages, GC/MS spectral data was combined with multivariate analysis. Unsupervised principal component analysis (PCA) was performed to check the clustering trends of features by reduction of dimensionality. A total of 9368 features which were extracted from data preprocessing by XCMS Online, were then analyzed by PCA. From the PCA score plot (R2X_(cum)_ = 0.807, Q2_(cum)_ = 0.624; Figure 2A), each of the samples tended to gather according to the developmental stage, into four clusters (stage one, stage two, stages three to six, and stages seven to eight). Stages one-two and stages seven-eight were clearly separated by PC1, explained by 34.5% of variance; while stages three-six were distinguished from other stages along PC2 by 21.0% of variance. Furthermore, a supervised partial least squares discriminant analysis (PLS-DA) model (R2X_(cum)_ = 0.630, R2Y_(cum)_ = 0.361, Q2_(cum)_ = 0.222; Figure 2B) was used to reveal the responsible metabolites for discrimination of developmental stages. The selection of variables represents the primary metabolites in the PLS-DA score plot based on the VIP value (>1.0) and *p*-value (<0.05). The identification of metabolites was examined on the basis of retention times, retention indices of reported metabolites [22,23,24,25,26,27,28], and mass fragmentation patterns, with reference to the National Institute of Standards and Technology (NIST) library. 

A total of 32 metabolites were selected and putatively identified as significant markers to discriminate between the eight developmental stages of *G. lingzhi*. A heat map analysis was applied to compare the relative levels of discriminant metabolites, on the basis of the corresponding peak area (Figure 2C). The discriminant markers were mostly primary metabolites including six organic acids, two amino acids, six sugars and polyols, seven fatty acids, three fatty alcohols, a phospholipid, and a nucleobase. In addition, six metabolites including two steroids were also selected (Table S1).

A vast majority of significantly different metabolites in the different developmental stages of *G. lingzhi* were observed in relatively greater quantities at stage one, including a sugar, fructose (**15**), polyols such as glycerol (**6**), mannitol (**16**), and myo-inositol (**19**); a phospholipid, glycerol 3-phosphate (**26**); organic acids such as phosphoric acid (**5**), succinic acid (**7**), malic acid (**8**), glutamic acid (**9**), and citric acid (**13**); fatty acids such as myristic acid (**14**), pentadecanoic acid (**17**), palmitic acid (**18**), linoleic acid methyl ester (**20**), margaric acid (**21**), linoleic acid (**22**), and lignoceric acid (**27**); a fatty alcohol, 5-nonanol (**2**); a nucleoside, 5-methyluridine (**23**); and other metabolites such as azelaic acid (**12**), chrysophanol (**26**), ergosterol (**31**), and phthalic acid ester (**24**) (Figure 2B). Pentadecanoic acid, linoleic acid, and 5-nonanol were also found in relatively higher level at stage two in addition to a polyamine, putrescine (**3**).

The following metabolites were detected in relatively higher-levels during stages three to six: a sugar, trehalose (**25**) which increased until stage eight; fatty alcohols such as tricosyl alcohol (**28**) and lignoceryl alcohol (**29**); steroids such as stellasterol (**32**) and ergosterol; and putrescine. Moreover, the levels of citric acid, 5-nonanol, glycerol 3-phosphate, and ethanolamine (**4**) were higher at stages three to four. Additionally, the quantities of glycerol and phosphoric acid peaked at stage three, while the levels of chrysophanol and eicosamethylcyclodecasiloxane (**30**) peaked at stage four.

The quantities of eicosamethylcyclodecasiloxane escalated at stages four to eight, while lactic acid (**1**) was found to be slightly higher at stages five to six. Xylitol (**10**), a sugar alcohol, and citric acid were found to be present at great quantities during stages seven to eight, compared to other developmental stages of *G. lingzhi*. Furthermore, the level of 5-methyluridine was found to be highest at stages six to seven. Interestingly, all of the metabolites which were found in greater quantities at stage one, except azelaic acid, chrysophanol, and phthalic acid ester, were also present in higher levels at stage seven.

### 2.2. Secondary Metabolite Profiling of G. lingzhi at the Eight Developmental Stages by LC/IT-TOF-MS

**Figure 3:**
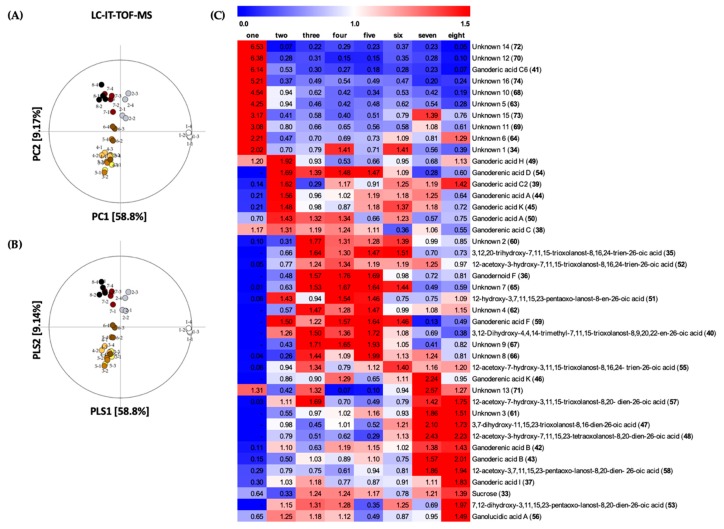
(**A**) PCA and (**B**) PLS-DA score plot of each *G. lingzhi* samples at the eight developmental stages analyzed by LC/IT-TOF-MS; (**C**) Heat maps of significantly different metabolites at the eight developmental stages of *G. lingzhi* from LC/IT-TOF-MS analysis. Each column represents the developmental stage, and the fold change of average peak area denoted by the number and color of heat scale.

The changes of secondary metabolites at the eight developmental stages of *G. lingzhi* were profiled by means of LC/IT-TOF-MS data, in combination with multivariate analysis. As shown in Figure 3A, stage one was clearly separated from the other stages in PC1 by 58.8% of variance; while stage two, seven, and eight alienated from stages three to six in PC2 by 9.17% of variance (R2X_(cum)_ = 0.680, Q2_(cum)_ = 0.615). Moreover, PLS-DA analysis was performed to select secondary metabolites that gave significant differences on the basis of VIP value (>1.0) and *p*-value (<0.05) (R2X_(cum)_ = 0.724, R2Y_(cum)_ = 0.412, Q2_(cum)_ = 0.325; Figure 3B). The identification of secondary metabolites was elucidated by comparing the retention time, molecular weight, and MS/MS fragmentation pattern of samples, to that of standard compounds or through references [29,30,31,32]. 

**Figure 4:**
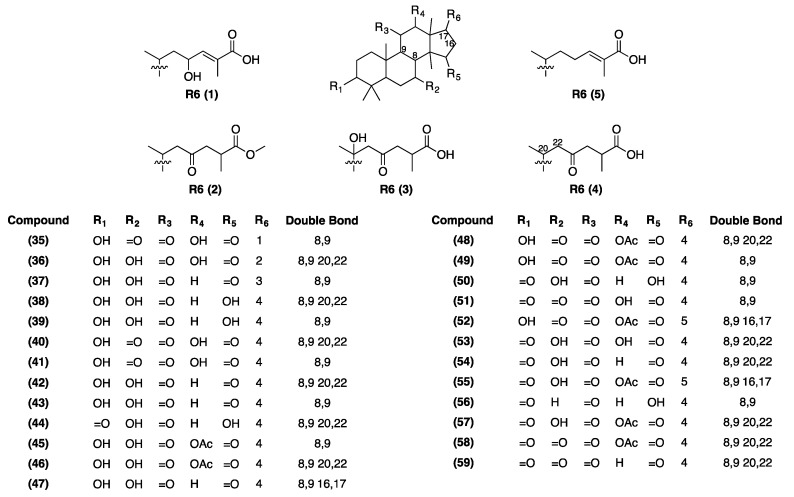
Chemical structures of selected discriminant metabolites from LC/IT-TOF-MS analysis.

A total of 42 metabolites, including 25 triterpenoids, sucrose, and 16 unidentified metabolites were selected for discrimination of *G. lingzhi* at eight developmental stage (Table S1). The variations of the quantity of secondary metabolites during the developmental stages of *G. lingzhi* has been illustrated in the heat map, as shown in Figure 3C. Ten metabolites were found to be present at their greatest quantities in stage one: unknown 1 (**34**), ganoderic acid C6 (**41**), unknown 5 (**63**), unknown 6 (**64**), unknown 10 (**68**), unknown 11 (**69**), unknown 12 (**70**), unknown 14 (**72**), unknown 15 (**73**), and unknown 16 (**74**). Furthermore, at stage two: ganoderenic acid C (**38**), ganoderic acid C2 (**39**), ganoderenic acid A (**44**), ganoderic acid K (**45**), ganoderic acid H (**49**), ganoderic acid A (**50**), and ganoderenic acid D (**54**) were found to be present in their highest levels. During stages three to six, the following metabolites were found in relatively higher levels: 3,12,20-trihydroxy-7,11,15-trioxolanost-8,16,24-trien-26-oic acid (**35**), 3,12-dihydroxy-4,4,14-trimethyl-7,11,15-trioxolanost-8,9,20,22-en-26-oic acid (**40**), 12-acetoxy-3-hydroxy-7,11,15-trioxolanost-8,16,24-trien-26-oic acid (**52**), ganoderenic acid F (**59**), unknown 2 (**60**), unknown 7 (**65**), unknown 8 (**66**), and unknown 9 (**67**). The levels of ganodernoid F (**36**), 12-hydroxy-3,7,11,15,23-pentaoxo-lanost-8-en-26-oic acid (**51**), unknown 4 (**62**) were present in relatively higher quantities during stages three to five, except **51** which was slightly lower at stage three. Furthermore, the quantities of sucrose (**33**), ganoderic acid I (**37**), ganoderenic acid B (**42**), ganoderic acid B (**43**), 3,7-dihydroxy-11,15,23-trioxolanost-8,16-dien-26-oic acid (**47**), 12-acetoxy-3-hydroxy-7,11,15,23-tetraoxolanost-8,20-dien-26-oic acid (**48**), 12-acetoxy-7-hydroxy-3,11,15-trioxolanost-8,20-dien-26-oic acid (**57**), 12-acetoxy-3,7,11,15,23-pentaoxo-lanost-8,20 -dien-26-oic acid (**58**), unknown 3 (**61**), and unknown 13 (**71**) were found in higher levels at stage seven to eight. In addition, the levels of ganoderenic acid K (**46**) and 12-acetoxy-7-hydroxy-3,11,15-trioxolanost-8,16,24-trien-26-oic acid (**55**) at stage six and at stage seven were found to be present in their highest levels, along with 7,12-dihydroxy-3,11,15,23-pentaoxo-lanost-8,20-dien-26-oic acid (**53**) and ganolucidic acid A (**56**) at stage eight. The structures of the discriminant metabolites can be seen in Figure 4.

### 2.3. Comparison of α-Glucosidase Inhibitory Activity of G. lingzhi at the Eight Developmental Stages

**Figure 5:**
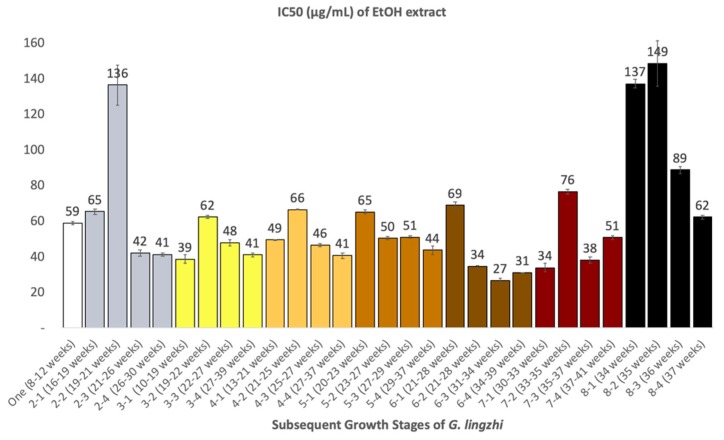
α-Glucosidase inhibitory activity (IC_50_ in μg/mL) of ethanol extract of *G. lingzhi* at different developmental stages.

The inhibitory activity of *G. lingzhi* at different developmental stages on α-glucosidase was investigated (Figure 5). Overall, ethanol extracts of *G. lingzhi* at every developmental stage showed stronger inhibitory effect on α-glucosidase enzymatic activity, compared to acarbose, a clinically approved α-glucosidase inhibitor which was used as a positive control in the present study, and which had an IC_50_ value of 347 ± 53.3 μg/mL (the data not shown in the figure). The average IC_50_ values of each stages in decreasing order were as follows: stage six (40 ± 0.9 μg/mL), stage three (47 ± 1.5 μg/mL), stage seven (50 ± 1.7 μg/mL), stage four (51 ± 0.8 μg/mL), stage five (52 ± 1.2 μg/mL), stage one (59 ± 0.9 μg/mL), stage two (71 ± 3.8 μg/mL), and stage eight (109 ± 4.6 μg/mL). Amongst all the developmental stages of *G. lingzhi*, the sample 6-3 which was harvested 31-34 weeks after the inoculation of the spawn was the strongest inhibitor, with an IC_50_ value of 27 ± 1.4 μg/mL.

## 3. Discussion

The untargeted metabolites profiling was performed for developmental stages of *G. lingzhi* using GC/MS and LC/IT-TOF-MS. From the PCA score plot (Figure 2A and Figure 3A), the profile of metabolites has been clustered into four clusters as follows: stage one, stage two, stages three-six, and stages seven-eight which corresponded to mycelial stage, primordia stage, bud-breaking of primordia stage to developmental stage, and mature stage to post-mature stage respectively.

Mycelia stage is a vegetative state in the initial formation of fruiting bodies of Basidiomycetes [33]. In this stage, we found great levels of sugars and polyols, organic acids, fatty acids, and fatty alcohols that might be related to the carbon source and the production of energy during exponential rate of mycelium expansion, for the formation of fruiting bodies [34,35]. Another reason could be because the mycelium greedily absorbs nutrient from the substrate, stores the nutrients, and releases it only when they are invaded [36]. Fructose and glycerol were reported as the best carbon source for mycelial development [34]. In addition to these sources of carbon, mannitol and myo-inositol have also been reported to exist in *Ganoderma* species at the mycelial stage in relatively higher concentrations [37]. Glycerol-3-phosphate is an intermediate related to glycolysis pathway; while malic acid, succinic acid, citric acid, and glutamic acid are well-known organic acids that are involved in TCA cycle for energy production in the mushroom cells [34,38]. The fatty acids such as myristic acid, pentadecanoic acid, palmitic acid, margaric acid, linoleic acid, and lignoceric acid are essential components of cell membrane and cell wall of mushroom, which functions as food reserves and cell protectors [34,39]. The relatively high quantity of 5-methyluridine, a nucleobase, at mycelial stage might be associated with its reproductive function in preparation for primordia formation [40]. As for the secondary metabolites, azelaic acid was reported as a weak competitive tyrosinase inhibitor which might contribute to the tyrosinase inhibitory activity of *G. lingzhi* extract [5,41]. For this reason, we suggest that azelaic acid also contributed to the white color of mycelia. Chrysophanol has been reported as a highly active inhibitor of pathogenic fungi on plants, by suppression of mycelial growth [42]. Similarly, ergosterol, a principle sterol which can be found only in fungi, was reported to have the ability to activate defense enzyme in plants, against pathogenic fungi [43]. Therefore, we imply that the biosynthesis of these compounds might be related to the survival function of *G. lingzhi* mycelia, and to provide a competitive advantage against other fungi. As for triterpenes, in agreement with the previous reports about its biosynthesis in *G. lingzhi* [44,45], our metabolomic data confirmed that only a few triterpenoids are produced at mycelial stage. Several lanostane-type triterpenoids such as ganoderic acid C6 (**41**), ganoderic acid H (**49**), and other unidentified metabolites were produced at highest quantities in this stage. Ganoderic acid A (**50**), ganoderic acid B (**43**), **41**, and **49** isolated from fruiting bodies of *G. lingzhi* were reported as anti-nociceptive components [46]. Moreover, the strong effect of acetone extract of *G. lingzhi* mycelium on anti-nociceptive activity has also been reported [47]. These facts show that there is a strong correlation between secondary metabolites contained in crude extracts of natural products and their biological activity. In addition, it is worthy to note that phthalic acid ester, which is usually used as plasticizer, was detected at all stages; particularly at the mycelial stage with the highest intensities. This might be related to the degradation of cultivation bags during the expansive growth of mycelia.

At the primordia stage, we observed that the levels of almost all of the selected markers of primary metabolites were decreased, compared to the mycelial stage, apart from pentadecanoic acid and linoleic acid, which were still above average levels. These fatty acids might be essential to support the formation of primordia. On the other hand, the levels of ethanolamine and putrescine were increased. The latter, at primordia stage, was noticed at its highest levels in comparison to other developmental stages. Previous studies about the role of putrescine in *G. lingzhi* shows that it is responsible for mushroom-like smell [48], and it influences the biosynthesis of ganoderic acid [49]. Thus, at this stage we found that more triterpenoids were produced i.e., ganoderenic acid C (**38**), ganoderic acid C2 (**39**), ganoderenic acid A (**44**), ganoderic acid K (**45**), ganoderic acid H (**49**), ganoderic acid A (**50**), and ganoderenic acid D (**54**). All of them except **54** showed strong inhibitory activity on angiotensin-converting enzyme inhibition assay [50]. **39** and **50** were reported to have inhibitory effect on the induction of Epstein–Barr Virus Early Antigen (EBV-EA) [51], while **39** and **44** exhibited aldose reductase inhibitory activity [10]. As mentioned earlier, several triterpenoids such as **43**, **49**, and **50** have anti-nociceptive activity at the mycelial stage, but, their relative levels were not as high as their quantities at the primordia stage. Hence, we suggest that the anti-nociceptive effect of the primordia stage must be due to their triterpenoid contents. Since the triterpenoids at primordia stage are capable of exhibiting various biological activities, specific *G. lingzhi* products can be prepared simply from the primordia itself.

Primordia eventually developed into immature fruiting bodies, and this period of development was divided into four stages (stages three to six), as can be seen in Figure 1. As for the bud-breaking stages, several changes of metabolites were observed, such as increased levels of sucrose, trehalose, glycerol, glycerol-3-phosphate, citric acid, and phosphoric acid from primordia stage. On the other hand, the levels of pentadecanoic acid and linoleic acid were decreased intensely. Trehalose has been reported to be the best source of carbohydrate for the formation of fruiting bodies, which is converted from glucose inside hyphae, as a food reserve [34]. Regarding the secondary metabolites at bud-breaking stage, rapid increase in the quantities of some metabolites such as ganodernoid F, **35**, **52**, **55**, **60**, **62**, **65**, **66**, **67**, and **71** were observed, while ganoderic acid C2 was reduced. We examined that the level of eicosamethylcyclodecasiloxane increased rapidly when the bud-breaking stage developed to early cap formation, whereas, glycerol intensities contrarily decreased. However, the correlation between the changes of eicosamethylcyclodecasiloxane or glycerol and the early cap formation remains unclear. Concerning secondary metabolites produced at this stage, great changes were observed as the increasing quantities of ganoderic acid C2, ganoderenic acid B, **34**, and **51**, and decreasing of ganoderic acid H, ganoderic acid I, **47**, **55**, **57**, and **71**. Furthermore, we checked the metabolites alteration during cap formation which was assigned by the observation of a white edge on the cap. At this stage, the level of citric acid declined intensively. Also, ganoderic acid A, ganoderenic acid K, **34**, **47**, **48**, **53**, and **56** were reduced immensely, but, a sharp increase was observed for **66**. The immature fruiting bodies was assigned by the yellow color of the edge of the cap. We noticed the sharp inclination of margaric acid at this stage, as well as 5-methyluridine, myo-inositol, and succinic acid. The increasing inclination of margaric acid might be related to the yellow color of the edge of the cap. As for 5-methyluridine, we suggest that it might be associated with the elevation of reproductive function prior to maturity stage [40].

In general, the levels of metabolites were increased at the mature stage and some of them such as ganoderenic acid K, **47**, **48**, **61**, and **71** were at their highest levels. In contrast, pentadecanoic acid, putrescine, tricosyl alcohol, lignoceryl alcohol, chrysophanol, stellasterol, and ergosterol were decreased. Ergosterol in particular could be considered as a suitable discriminant marker for mature stage, since its quantities declined greatly in comparison to immature stage. As for putrescine, due to its role in influencing the biosynthesis of triterpenes, its declining levels at the mature stage indicates the maturity of fruiting bodies. In mature stage, the spores will accumulate until the dispersion. We observed that at this stage, when the spores have been dispersed, the levels of all metabolites decreased, except eicosamethylcyclodecasiloxane, myristic acid, citric acid, and xylitol. Especially, xylitol was observed at its highest levels in this stage, compared to other developmental stages.

In order to demonstrate the correlation between biological activity and variability of metabolites during developmental stages of *Ganoderma lingzhi*, we performed alpha-glucosidase inhibitory assay as shown in Figure 5. We observed that immature stage of *G. lingzhi* gave the highest activity (lowest IC50 value) especially for the sample which was harvested 31–34 weeks after the inoculation of spawn. This inhibitory activity might be associated with the accumulation of active compounds at this stage. To find the responsible metabolites that gave higher activity, we performed Pearson’s correlation test between the 1/IC_50_ of each developmental stage and the peak area of the discriminant metabolites. As a result, several metabolites such as 34 and 35 gave medium positive correlation. Our current results show that immature stage correlates with the highest α-glucosidase inhibitory activity. This result also proves that the mature fruiting bodies are not always necessary for good quality *G. lingzhi* products; and the harvesting time of the fruiting bodies should depend on the specific purpose of *G. lingzhi* product.

## 4. Materials and Methods

### 4.1. Chemicals and Reagents

Deionized water was purified using an Ultrapure Water System RFU424CA (Advantec, Tokyo, Japan). LC-MS grade of methanol, acetonitrile, formic acid, dehydrated pyridine, sucrose, and acarbose were purchased from Fujifilm Wako Pure Chemical Corporation, Osaka, Japan. Methoxyamine hydrochloride was obtained from Aldrich (St Louis, Mo., USA). *N*-methyl-*N*-(trimethylsilyl)-trifluoroacetamide (MSTFA) + 1% trimethylchlorosilane (TMCS) was purchased from Thermo Scientific, Rockford, IL, USA. C_8_–C_20_ alkane analytical standard solution containing ~40 mg/L of each C_8_–C_20_ alkane in hexane was obtained from Fluka (Menlo Park, CA, USA). Triterpenoid standards of ganoderic acid A, ganoderic acid B, ganoderenic acid C, ganoderic acid C1, ganoderic acid C2, ganoderic acid C6, ganoderenic acid D, ganoderic acid I, and ganoderic acid K were purchased from Chem Faces Co., Ltd. (Wuhan, China). Ultrol grade HEPES was purchased from Calbiochem (San Diego, CA, USA). α-Glucosidase derived from yeast was obtained from Oriental Yeast Co. Ltd. (Tokyo, Japan).

### 4.2. Mushroom Materials and Sampling

*G. lingzhi* samples were received every week since February 14 to September 5, 2018 from Aso Biotech, a mushroom company located in Kumamoto, Japan. The spawn of *G. lingzhi* strain BMC9049 was inoculated into the substrate packed in heat-sealed cultivation bags, with microfilter windows. The substrate in each bag consisted of wood chips (hardwood) 1563 g, rice bran 93 g, wheat bran 69 g, and water 775 g, resulting in final moisture of 55% in the mushroom bed. The environmental condition of the cultivation site was maintained at the temperature 23–25 °C, humidity 80%, and air circulation for more than 12 h/day. The sample harvests were done randomly.

For the identification of different developmental stages, fruiting body samples were sent to Dr. Hiroto Suhara, a mushroom expert from Miyazaki Prefectural Wood Utilization Research Center, Japan. Eight developmental stages of *G. lingzhi* fruiting bodies were categorized on the basis of morphological changes; starting from mycelium stage to post-mature stage. *G. lingzhi* samples were freeze dried at approximately −50 °C temperature and 50 mmHg pressure. Sampling of each developmental stages were made by pooling the samples based on morphological observations into four levels, according to their age (in weeks), wherein each level consists of more than three fruiting bodies (*n* > 3). The detailed information regarding *G. lingzhi* samples used are listed in Table 1. The dried samples were pulverized and then keep in a dark and dry place at room temperature prior to extraction.

### 4.3. Metabolite Extraction and Sample Preparation

Pulverized fruiting bodies of *G. lingzhi* from each stage (500 mg) were transferred to a 15 mL High-Clarity Polypropylene Conical Tube (Falcon, Corning Science Mexico SA de CV, Tamaulipas, Mexico), and extracted by sonication for 30 min using 7 mL of ethanol. After centrifugation for 10 min at 2058× *g*, the supernatant was separated. The same extraction process was repeated by adding 3 mL of ethanol. The supernatants from both extractions were combined respectively, and then filtered, and evaporated in a rotary evaporator under vacuum at 45 °C. The dried extract was then reconstituted in methanol to make a 10 mg/mL solution. After being vortexed, the solution was filtered through 0.20 µm PTFE syringe driven filter unit (Millex-LG, Millipore, Tokyo, Japan), and then analyzed by LC/IT-TOF-MS and GC/MS. The sample oximation for GC/MS analysis was performed by incubating the dried sample extracts with 50 μL of methoxyamine hydrochloride in dry pyridine (20 mg/mL) at 30 °C for 90 min. Subsequently, the silylation step was carried out by adding 50 μL of MSTFA + 1%TMCS to the reaction mixture with 30 min incubation at 37 °C.

### 4.4. GC/MS Analysis

A GC/MS analysis was performed using a 7890A GC-5975C inert MSD (with Triple Axis Detector) system (Agilent Technologies, Palo Alto, CA). One microliter of sample was injected into the HP-5MS Agilent capillary column (30 m × 0.25 mm i.d.; 0.25 μm thickness) using an auto-injector with a split ratio of 1:10. Helium was used as a carrier gas at a flow rate of 1.5 mL/min. The inlets and MS source temperatures were maintained at 250 and 230 °C respectively. The oven temperature was maintained at 75 °C for 2 min and ramped to 300 °C at a rate of 10 °C/min, then held at 300 °C for 10 min. Data were acquired in full scan from 50 to 800 m/z. The metabolites were putatively identified based on library searches in the NIST database. The level of confidence for the identification of metabolites was based upon Metabolomics Standard Initiative (MSI) of the Metabolomics Society [52].

### 4.5. LC/IT-TOF-MS Analysis

Liquid chromatographic separation and mass spectrometric detection were achieved by employing UFLC coupled with IT-TOF-MS via electrospray ionization (ESI) interface (Shimadzu, Kyoto, Japan). The analytical column was ZORBAX Eclipse plus C18 RRHT (3.0 mm i.d. × 100 mm, 1.8 µm) equipped with Eclipse plus C18 guard column (3.0 mm i.d. × 5 mm, 1.8 µm). The automatic sampler was maintained at 4 °C and the injection volume was 2 µL. The gradient elution was performed using a 40 min method, at a flow rate of 0.43 mL/min, with the mobile phase containing water with 0.1% formic acid (mobile phase A) and acetonitrile with 0.1% formic acid (mobile phase B). In the first 26 min, mobile phase B was increased linearly from 5% to 100%. Then, mobile phase B was kept at 100% for 9 min. At 31–32 min, sodium trifluoroacetic (NaTFA) was injected into the mass system for mass calibration. At 35.1 min, mobile phase B was adjusted to 5% for equilibration for 5 min. Mass spectra in both positive and negative ionization modes were obtained simultaneously in a full-scan operation with a scan range of 100–1000, 50–650, and 50–300 *m*/*z* for MS^1^, MS^2^, and MS^3^ respectively by switching the interface voltage between 4.5 kV and 3.5 kV in each 0.1 s. The CID collision energy of MS^2^ and MS^3^ were set to 50 and 75%, respectively. The flow rate of the nebulizing gas (N_2_) was 1.5 L/min. The temperatures of the curved desorption line and the heat block were both 200 °C, and the microchannel plate detector voltage was set to 1.70 kV. The pressure of the drying gas (N_2_) was 198 kPa, and the ion accumulation time was set to 10 ms. Mass spectra and chromatograms were acquired and processed with LC/MS solution version 3.0 (Shimadzu). The metabolites were positively identified using standard compounds. In the absence of the standard compounds, a tentative identification was made by comparing the MS and MS^n^ spectra of metabolites, reported in previously published research. The confidence level of metabolite annotations was also based upon MSI [52].

### 4.6. α-Glucosidase Inhibitory Activity

The α-glucosidase activity was carried out according to a previously described method with minor modifications [9]. A 100 µL portion of α-glucosidase (5 units/mL) in 0.15 M HEPES buffer was added to the mixture of 100 µL DMSO with or without (control) samples and 100 µl of sucrose in 0.15 M HEPES buffer, and then incubated at 37 °C for 30 min. After incubation, the reaction was ended by heating at 100 °C for 10 min. The formation of glucose was determined by means of the glucose oxidase method using a BF-5S Biosensor (Oji Scientific Instrument, Hyogo, Japan).

### 4.7. Data Processing and Multivariate Analysis

GC/MS raw data files were converted to computable document form (CDF) format (*.cdf) using the Enhanced Data analysis (Agilent). The converted GC/MS data files were pre-processed for peak alignment, retention time correction, and peak intensity calculation using a web-based software, XCMS Online (xcmsonline.scripps.edu). A default Centwave method for single quadrupole, “GC/Single Quad (centWave)”, was selected as the parameter set [53]. The parameters were as follows: maximal tolerated m/z deviation, 100 ppm; signal/noise threshold, 6; mzdiff, 0.01; integration methods, 1; prefilter peaks, 3; prefilter intensity, 100; mzwid, 0.25; minfrac, 0.5; and bandwidth, 10. After processing, a data matrix containing retention times, masses, and normalized peak intensities were obtained in an Excel (Microsoft, Redmond, WA, USA) format and was analyzed further with multivariate software.

The obtained data from LC/IT-TOF-MS were processed by Profiling Solution version 1.1 (Shimadzu) for peak deconvolution and alignment. The method parameters were as follows: width (5 s), slope (2000/min), retention time range (0.5–30 min), ion *m*/*z* tolerance (20 mDa), ion retention time tolerance (0.5 min), and ion intensity threshold (10,000 counts). After completing the integration parameters, a report of peaks based on areas, retention time and *m*/*z* was generated for each sample. Signals of different samples were considered to be similar when they simultaneously fulfilled both retention time (0.5 min tolerance) and *m*/*z* value (30 mDa tolerance) criteria. The resulting data were exported to Excel for multivariate analysis.

Multivariate statistical analysis was performed using SIMCA (version 15.0.2, Umetrics, Umea, Sweden). PCA and PLS-DA models were used to compare the changes of metabolites during developmental stages of *G. lingzhi*. The data sets were pareto scaled without transformation prior to PCA and PLS-DA modelling. The PCA model was evaluated with statistical parameters, including the R2X which explained variance of X-data, and the Q2 which illustrated the predicted variance. In PLS-DA model, the R2X and R2Y represent fraction of the variance of the X and Y matrix variables explained by the model. Q2 represented the predictive capacity of the model. R2X(cum) and R2Y(cum) is cumulative fraction of sum of squares of X and Y explained by the current component, respectively. Metabolites with variable importance as determined by a projection (VIP) value of >1.0 and a *p*-value of <0.05 were selected. Significant differences were tested by one-way analysis of variance (ANOVA) using SPSS Statistics 24 (SPSS Inc., Chicago, IL, USA). Differences in alpha-glucosidase inhibitory activity were analyzed by one-way ANOVA and Duncan’s multiple-range test using SPSS Statistics 24 (SPSS Inc.). Pairwise correlations between secondary metabolites and alpha-glucosidase inhibitory activity were calculated by Pearson’s correlation coefficient test using Excel.

## 5. Conclusions

MS-based untargeted metabolomics was carried out on the developmental stages of *G. lingzhi*. We confirmed that there are significant changes in the primary and secondary metabolites during the developmental stages of *G. lingzhi*. Moreover, we found that the state of the metabolites during various developmental stages of *G. lingzhi* was associated with the morphological changes. Two metabolites, **34** and **35**, were found to have potential contribution to the α-glucosidase inhibitory activity. The list of selected marker metabolites might be useful for discrimination of developmental stages. It might not only be helpful in explaining the variability in the quality of *G. lingzhi* products, but can also be applicable for increasing cultivation efficiency through selection of optimum harvesting time of *G. lingzhi*. Our current findings may provide practical information for more comprehensive targeted analysis of selected markers in *G. lingzhi*, for prospective studies in the future.

## Figures and Tables

**Table 1:** List of harvested *G. lingzhi* (BMC9049) at different developmental stages after spawn inoculation.

Developmental Stages	Stage One ^a^	Stage Two ^b^	Stage Three ^c^	Stage Four ^d^	Stage Five ^e^	Stage Six ^f^	Stage Seven ^g^	Stage Eight ^h^
Sample label (age in weeks)	1-1 (8–12)	2-1 (16–19)	3-1 (10–19)	4-1 (13–21)	5-1 (20–23)	6-1 (21–28)	7-1 (30–33)	8-1 (34)
	1-2 (8–12)	2-2 (19–21)	3-2 (19–22)	4-2 (21–25)	5-2 (23–27)	6-2 (28–31)	7-2 (33–35)	8-2 (35)
	1-3 (8–12)	2-3 (21–26)	3-3 (22–27)	4-3 (25–27)	5-3 (27–29)	6-3 (31–34)	7-3 (35–37)	8-3 (36)
	1-4 (8–12)	2-4 (26–30)	3-4 (27–39)	4-4 (27–37)	5-4 (29–37)	6-4 (34–39)	7-4 (37–41)	8-4 (37)

^a^ mycelia; ^b^ primordia; ^c^ bud-breaking, brown stipe; ^d^ early cap formation; ^e^ cap formation, white edge; ^f^ immature stage, yellow edge; ^g^ mature stage, spore not dispersed; ^h^ post-mature stage, after spore dispersed.

## Supplementary Materials

The following are available online. Figure S1: Principle Component Analysis (PCA) loading plot of metabolites derived from GC/MS data sets of *Ganoderma lingzhi* at eight developmental stages, Figure S2: Principle Component Analysis (PCA) loading plot of metabolites derived from LC/IT-TOF-MS data sets of *Ganoderma lingzhi* at eight developmental stages, Table S1: Tentatively identified chemical markers at eight developmental stages of *G. lingzhi* analyzed by GC/MS and LC/IT-TOF-MS.

## References

[1] Cheung P.C.K. (2013). Mini-review on edible mushrooms as source of dietary fiber: Preparation and health benefits. Food Sci. Hum. Wellness.

[2] Wachtel-Galor S., Tomlinson B., Benzie I.F.F. (2004). *Ganoderma lucidum* (‘Lingzhi’), a Chinese medicinal mushroom: Biomarker responses in a controlled human suppl. study. Br. J. Nutr..

[3] Wachtel-galor S., Szeto Y., Tomlinson B., Benzie I.F. (2004). *Ganoderma lucidum* (‘Lingzhi’); acute and short-term biomarker response to supplementation. Int. J. Food Sci. Nutr..

[4] Satria D., Amen Y., Niwa Y., Ashour A., Allam A.E., Shimizu K. (2019). Lucidumol D, a new lanostane-type triterpene from fruiting bodies of Reishi (*Ganoderma lingzhi*). Nat. Prod. Res..

[5] Chien C.-C., Tsai M.-L., Chen C.-C., Chang S.-J., Tseng C.-H. (2008). Effects on tyrosinase activity by the extracts of *Ganoderma lucidum* and related mushrooms. Mycopathologia.

[6] Kim J.-W., Kim H.-I., Kim J.-H., Kwon O.-C., Son E.-S., Lee C.-S., Park Y.-J. (2016). Effects of ganodermanondiol, a new melanogenesis inhibitor from the medicinal mushroom *Ganoderma lucidum*. Int. J. Mol. Sci..

[7] Fatmawati S., Shimizu K., Kondo R. (2010). Ganoderic acid Df, a new triterpenoid with aldose reductase inhibitory activity from the fruiting body of *Ganoderma lucidum*. Fitoterapia.

[8] Fatmawati S., Shimizu K., Kondo R. (2010). Inhibition of aldose reductase in vitro by constituents of *Ganoderma lucidum*. Plant. Med..

[9] Fatmawati S., Shimizu K., Kondo R. (2011). Ganoderol B: A potent α-glucosidase inhibitor isolated from the fruiting body of *Ganoderma lucidum*. Phytomedicine.

[10] Fatmawati S., Shimizu K., Kondo R. (2011). Structure–activity relationships of ganoderma acids from *Ganoderma lucidum* as aldose reductase inhibitors. Bioorg. Med. Chem. Lett..

[11] Wachtel-Galor S., Yuen J., Buswell J.A., Benzie I.F.F., Benzie I.F.F., Wachtel-Galor S. (2011). Ganoderma lucidum (Lingzhi or Reishi): A Medicinal Mushroom. Herbal Medicine: Biomolecular and Clinical Aspects.

[12] Hapuarachchi KK K. (2018). Current status of global Ganoderma cultivation, products, industry and market. Mycosphere.

[13] Liu J., Kurashiki K., Fukuta A., Kaneko S., Suimi Y., Shimizu K., Kondo R. (2012). Quantitative determination of the representative triterpenoids in the extracts of *Ganoderma lucidum* with different growth stages using high-performance liquid chromatography for evaluation of their 5α-reductase inhibitory properties. Food Chem..

[14] O’Gorman A., Barry-Ryan C., Frias J.M. (2012). Evaluation and identification of markers of damage in mushrooms (*Agaricus bisporus*) postharvest using a GC/MS metabolic profiling approach. Metabolomics.

[15] Woldegiorgis A.Z., Abate D., Haki G.D., Ziegler G.R. (2015). LC-MS/MS based metabolomics to identify biomarkers unique to *Laetiporus sulphureus*. Int. J. Nutr. Food Sci..

[16] Putri S.P., Fukusaki E. (2014). Mass Spectrometry-Based Metabolomics: A Practical Guide.

[17] Lei Z., Huhman D.V., Sumner L.W. (2011). Mass spectrometry strategies in metabolomics. J. Biol. Chem..

[18] t’Kindt R., Morreel K., Deforce D., Boerjan W., Van Bocxlaer J. (2009). Joint GC–MS and LC–MS platforms for comprehensive plant metabolomics: Repeatability and sample pre-treatment. J. Chromatogr. B.

[19] Lee S., Do S.-G., Kim S.Y., Kim J., Jin Y., Lee C.H. (2012). Mass spectrometry-based metabolite profiling and antioxidant activity of aloe vera (*Aloe barbadensis Miller*) in different growth stages. J. Agric. Food Chem..

[20] Lombardo V.A., Osorio S., Borsani J., Lauxmann M.A., Bustamante C.A., Budde C.O., Andreo C.S., Lara M.V., Fernie A.R., Drincovich M.F. (2011). Metabolic profiling during peach fruit development and ripening reveals the metabolic networks that underpin each developmental stage. Plant Physiol..

[21] Nakagawa T., Zhu Q., Tamrakar S., Amen Y., Mori Y., Suhara H., Kaneko S., Kawashima H., Okuzono K., Inoue Y. (2018). Changes in content of triterpenoids and polysaccharides in *Ganoderma lingzhi* at different growth stages. J. Nat. Med..

[22] Isidorov V.A., Lech P., Żółciak A., Rusak M., Szczepaniak L. (2008). Gas chromatographic–mass spectrometric investigation of metabolites from the needles and roots of pine seedlings at early stages of pathogenic fungi. Armillaria ostoyae attack. Trees.

[23] Birkemeyer C., Kopka J., Nikolau B.J., Wurtele E.S. (2007). Design of Metabolite Recovery by Variations of the Metabolite Profiling Protocol. Proceedings of the Concepts in Plant Metabolomics.

[24] Erxleben A., Gessler A., Vervliet-Scheebaum M., Reski R. (2012). Metabolite profiling of the moss *Physcomitrella patens* reveals evolutionary conservation of osmoprotective substances. Plant Cell Rep..

[25] Isidorov V.A., Kotowska U., Vinogorova V.T. (2005). GC identification of organic compounds based on partition coefficients of their TMS derivatives in a hexane-acetonitrile system and retention indices. Anal. Sci..

[26] Kim J.-S., Chung H.Y. (2009). GC-MS analysis of the volatile components in dried boxthorn (*Lycium chinensis*) fruit. J. Korean Soc. Appl. Biol. Chem..

[27] Yaşar A., Üçüncü O., Güleç C., İnceer H., Ayaz S., Yayl N. (2005). GC-MS analysis of chloroform extracts in flowers, stems, and roots of *Tripleurospermum callosum*. Pharm. Biol..

[28] Radulović N.S., Đorđević N.D. (2011). Steroids from poison hemlock (*Conium maculatum* L.): A GC-MS analysis. J. Serbian Chem. Soc..

[29] Qian Z., Zhao J., Li D., Hu D., Li S. (2012). Analysis of global components in *Ganoderma* using liquid chromatography system with multiple columns and detectors. J. Sep. Sci..

[30] Cheng C.-R., Yang M., Wu Z.-Y., Wang Y., Zeng F., Wu W.-Y., Guan S.-H., Guo D.-A. (2011). Fragmentation pathways of oxygenated tetracyclic triterpenoids and their application in the qualitative analysis of *Ganoderma lucidum* by multistage tandem mass spectrometry. Rapid Commun. Mass Spectrom..

[31] Wu L., Liang W., Chen W., Li S., Cui Y., Qi Q., Zhang L. (2017). Screening and analysis of the marker components in *Ganoderma lucidum* by HPLC and HPLC-MSn with the aid of chemometrics. Molecules.

[32] Yang M., Wang X., Guan S., Xia J., Sun J., Guo H., Guo D. (2007). Analysis of triterpenoids in *Ganoderma lucidum* using liquid chromatography coupled with electrospray ionization mass spectrometry. J. Am. Soc. Mass Spectrom..

[33] Kües U., Liu Y. (2000). Fruiting body production in basidiomycetes. Appl. Microbiol. Biotechnol..

[34] Miles P.G., Chang S.-T., Chang S.-T. (2004). Mushrooms: Cultivation, Nutritional Value, Medicinal Effect, and Environmental Impact.

[35] Trinci A.P.J. (1974). A study of the kinetics of hyphal extension and branch initiation of fungal mycelia. Microbiology.

[36] Boddy L., Watkinson S.C. (1995). Wood decomposition, higher fungi, and their role in nutrient redistribution. Can. J. Bot..

[37] Tseng Y.-H., Lee Y.-L., Li R.-C., Mau J.-L. (2005). Non-volatile flavour components of *Ganoderma tsugae*. Food Chem..

[38] Moore D. (2002). Fungal Morphogenesis.

[39] Etten J.L.V., Gottlieb D. (1965). Biochemical changes during the growth of fungi. J. Bacteriol..

[40] Park Y.J., Jung E.S., Singh D., Lee D.E., Kim S., Lee Y.W., Kim J.-G., Lee C.H. (2017). Spatial (cap & stipe) metabolomic variations affect functional components between brown and white beech mushrooms. Food Res. Int..

[41] Kim Y.-J., Uyama H. (2005). Tyrosinase inhibitors from natural and synthetic sources: Structure, inhibition mechanism and perspective for the future. CmlsCell. Mol. Life Sci..

[42] Choi G.J., Lee S.-W., Jang K.S., Kim J.-S., Cho K.Y., Kim J.-C. (2004). Effects of chrysophanol, parietin, and nepodin of *Rumex crispus* on barley and cucumber powdery mildews. Crop Prot..

[43] Shao S., Hernandez M., Kramer J.K.G., Rinker D.L., Tsao R. (2010). Ergosterol profiles, fatty acid composition, and antioxidant activities of button mushrooms as affected by tissue part and developmental stage. J. Agric. Food Chem..

[44] Liang W.-Q., Zhang D.-B., Wang N., Wang C.-G., Pan Y.-J. (2007). Cloning and characterization of squalene synthase (SQS) gene from *Ganoderma lucidum*. J. Microbiol. Biotechnol..

[45] Zhao M.W., Zhong J.Y., Liang W.Q., Wang N., Chen M.J., Zhang D.B., Pan Y.J., Jong S.C. (2004). Analysis of squalene synthase expression during the development of *Ganoderma lucidum*. J. Microbiol. Biotechnol..

[46] Koyama K., Imaizumi T., Akiba M., Kinoshita K., Takahashi K., Suzuki A., Yano S., Horie S., Watanabe K., Naoi Y. (1997). Antinociceptive components of *Ganoderma lucidum*. Plant. Med..

[47] Han C. (2009). A Comparison of antinociceptive activity of mycelial extract from three species of fungi of basidiomycetes. Open Complement. Med. J..

[48] Costa R., De Grazia S., Grasso E., Trozzi A. Headspace-Solid-Phase Microextraction-Gas Chromatography as Analytical Methodology for the Determination of Volatiles in Wild Mushrooms and Evaluation of Modifications Occurring during Storage. https://www.hindawi.com/journals/jamc/2015/951748/abs/.

[49] Wu C.-G., Tian J.-L., Liu R., Cao P.-F., Zhang T.-J., Ren A., Shi L., Zhao M.-W. (2017). Ornithine decarboxylase-mediated production of putrescine influences ganoderic acid biosynthesis by regulating reactive oxygen species in *Ganoderma lucidum*. Appl. Env. Microbiol..

[50] Hai-Bang T., Shimizu K. (2015). Structure–activity relationship and inhibition pattern of reishi-derived (*Ganoderma lingzhi*) triterpenoids against angiotensin-converting enzyme. Phytochem. Lett..

[51] Akihisa T., Nakamura Y., Tagata M., Tokuda H., Yasukawa K., Uchiyama E., Suzuki T., Kimura Y. (2007). anti-inflammatory and anti-tumor-promoting effects of triterpene acids and sterols from the fungus *Ganoderma lucidum*. Chem. Biodivers..

[52] Blaženović I., Kind T., Ji J., Fiehn O. (2018). Software tools and approaches for compound identification of LC-MS/MS data in metabolomics. Metabolites.

[53] Chi L., Bian X., Gao B., Tu P., Lai Y., Ru H., Lu K. (2018). Effects of the artificial sweetener neotame on the gut microbiome and fecal metabolites in mice. Molecules.

