# Supplementary material for: Mass Spectrometry-Based Untargeted Metabolomics and α-Glucosidase Inhibitory Activity of Lingzhi (Ganoderma lingzhi) During the Developmental Stages
Source: Molecules. 2019 May 29;24(11):2044. doi: 10.3390/molecules24112044 (PMC6600326; doi:10.3390/molecules24112044)
Supplement: Supplementary file 1 [file molecules-24-02044-s001.pdf]

Supplementary material

Mass Spectrometry-Based Untargeted Metabolomics and α-Glucosidase Inhibitory Activity of Lingzhi (*Ganoderma lingzhi*) During the Developmental Stages

Dedi Satria [1,2], Sonam Tamrakar [1], Hiroto Suhara [3], Shuhei Kaneko [4,†] and Kuniyoshi Shimizu [1,*]

[1] Division of Systematic Forest and Forest Products Sciences, Department of Agro-Environmental Sciences, Graduate School of Bioresource and Bioenvironmental Sciences, Kyushu University, Fukuoka 812-8581, Japan; dedi.satria@umsb.ac.id or dedi.satoria@gmail.com (D.S.); tamrakar.snm@gmail.com (S.T.)

[2] Faculty of Health and Sciences, Muhammadiyah University of Sumatera Barat, Bukittinggi 26181, Indonesia

[3] Miyazaki Prefectural Wood Utilization Research Center, Miyazaki 885-0037, Japan; suhara-hiroto@pref.miyazaki.lg.jp

[4] Fukuoka Prefecture Forest Research & Extension Center, 818-8549 Fukuoka, Japan; shu-k@kir.biglobe.ne.jp

* Correspondence: shimizu@agr.kyushu-u.ac.jp; Tel.: +81-92-802-4675

† (S.K. formerly worked in Fukuoka Prefecture Forest Research & Extension Center)

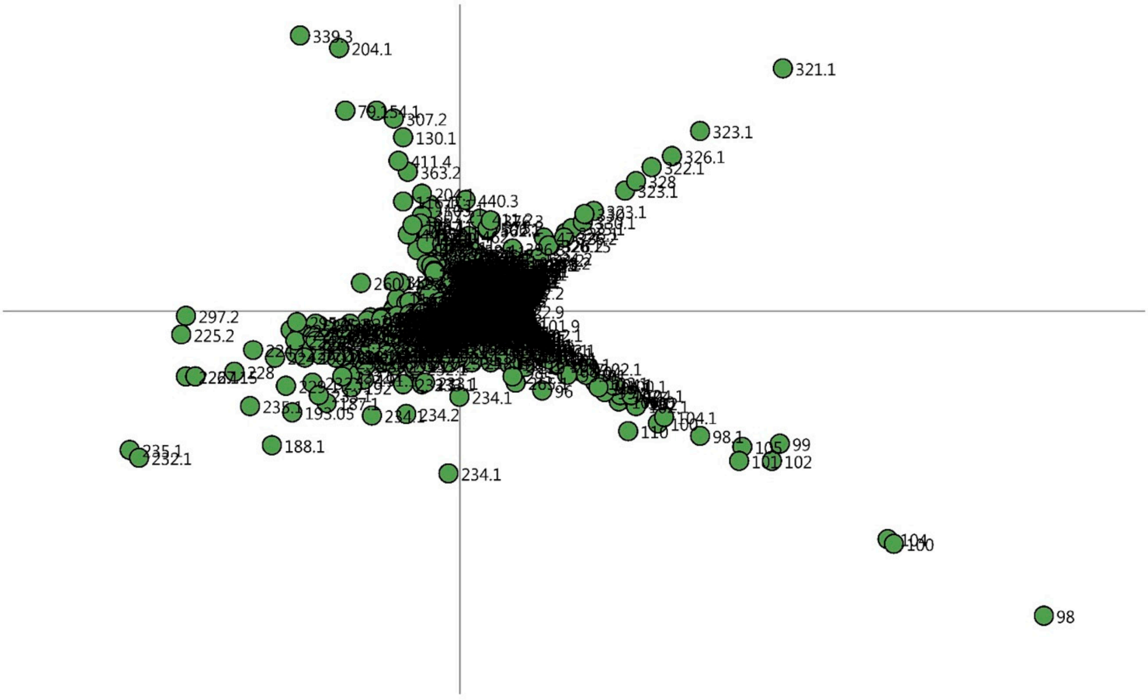

Figure S1. Principle Component Analysis (PCA) loading plot of metabolites derived from GC/MS data sets of *Ganoderma lingzhi* at eight developmental stages.

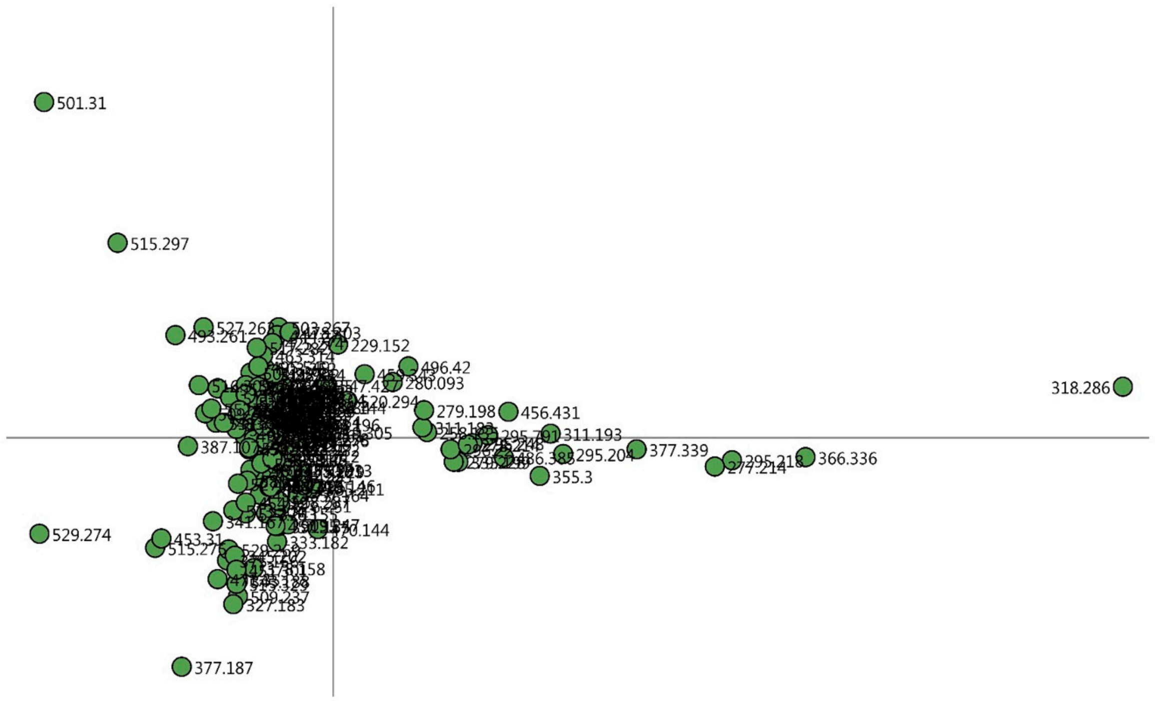

Figure S2. Principle Component Analysis (PCA) loading plot of metabolites derived from LC/IT-TOF-MS data sets of *Ganoderma lingzhi* at eight developmental stages.

Table S1. Tentatively identified chemical markers at eight developmental stages of *G. lingzhi* analyzed by GC/MS and LC/IT-TOF-MS.

ID[a]	RT[b] (min)	RI[c] Exp.	Tentative identification	|ΔRI|	Match correlation	Elemental Formula	Exact Mass	m/z [M-H]-	Mass error |Δppm|	Mass Fragmentation	ID level[d]	Ref.
**1***	5.04	1056	Lactic acid	10	955					147, **73**, 117, 191, 148, 190, 66, 149, 133, 118	2	[1]
**2***	5.16	1064	5-Nonanol	N/A	605					**56**, 73, 159, 186, 75, 143, 145, 57, 59, 158	3	
**3***	6.48	1151	Putrescine	N/A	752					174, **73**, 175, 216, 86, 142, 59, 176, 100, 160	3	
**4***	7.62	1227	Ethanolamine	42	876					**174**, 73, 175, 147, 100, 86, 176, 59, 130, 133	3	[2]
**5***	8.20	1268	Phosphoric acid	14	947					**299**, 300, 73, 314, 301, 133, 211, 283, 207, 315	2	[2]
**6***	8.21	1270	Glycerol	12	940					73, 147, 205, 117, **103**, 133, 218, 206, 148, 74	2	[2]
**7***	8.82	1310	Succinic acid	11	957					**147**, 73, 247, 75, 148, 149, 55, 129, 74, 172	2	[1]
**8***	11.11	1483	Malic acid	3	944					**73**, 147, 233, 245, 133, 148, 189, 75, 190, 74	2	[3]
**9***	12.70	1615	Glutamic acid	11	918					246, **73**, 247, 128, 147, 75, 248, 156, 348, 230	2	[3]
**10***	13.81	1716	Xylitol	1	868					**73**, 147, 103, 205, 307, 129, 319, 204, 189, 148	2	[3]
**11***	14.28	1756	Glycerol-3-phosphate	19	956					**357**, 299, 147, 358, 315, 300, 211, 445, 370, 359	2	[2]
**12***	14.68	1792	Azelaic acid	15	825					**116**, 73, 75, 317, 243, 147, 217, 204, 201, 129, 230	2	[1]
**13***	14.89	1812	Citric acid	8	793					**273**, 147, 363, 347, 274, 375, 465, 211, 305, 364	2	[3]
**14***	15.24	1846	Myristic acid	4	878					285, **117**, 73, 75, 129, 286, 145, 55, 131	2	[4]
**15***	15.54	1873	Fructose	1	824					**219**, 73, 217, 103, 307, 147, 218, 308, 74, 117, 133	2	[3]
**16***	16.03	1922	Mannitol	6	959					**73**, 319, 205, 147, 217, 103, 320, 307, 321, 117	2	[2]
**17***	16.25	1945	Pentadecanoic acid	4	883					299, 117, 73, 75, 132, **129**, 145, 300, 55, 131	2	[2]
**18***	17.21	2044	Palmitic acid	6	961					**313**, 117, 73, 75, 132, 129, 145, 314, 55, 131	2	[5]
**19***	17.57	2082	Myo-Inositol	9	928					305, 73, **217**, 147, 318, 191, 306, 204, 319, 265	2	[3]
**20***	17.67	2092	Linoleic acid methyl ester	2	841					67, 81, **95**, 55, 82, 79, 68, 96, 54, 109	2	[5]
**21***	18.12	2141	Margaric acid	8	902					**327**, 117, 73, 75, 132, 129, 145, 328, 55, 342	2	[1]
**22***	18.83	2211	Linoleic acid	1	874					75, **73**, 337, 67, 81, 95, 79, 55, 82, 96	2	[1]
**23***	20.26	2388	5-Methyluridine	N/A	719					73, 327, 147, 328, 75, 129, **217**, 103, 149, 74	3	

Table S1. *Cont.*

**24***	21.37	2529	Phthalic acid, bis(2-ethylhexyl) ester	10	881					149, 167, **57**, 71, 70, 150, 279, 113, 104, 83	2	[6]
**25***	22.90	2735	Trehalose	9	955					**361**, 73, 362, 191, 217, 147, 363, 204, 103, 271	2	[3]
**26***	23.18	2774	Chrysophanol	N/A	774					**383**, 147, 384, 57, 103, 385, 83, 55, 97, 485	3	
**27***	23.61	2834	Lignoceric acid	1	810					117, 73, 425, **75**, 132, 145, 129, 57, 55, 426	2	[3]
**28***	23.87	2871	Tricosyl alcohol	N/A	784					**397**, 398, 147, 129, 57, 75, 103, 399, 83, 55	3	
**29***	24.54	2968	Lignoceryl alcohol	N/A	790					**411**, 73, 412, 147, 129, 57, 413, 75, 103, 83	3	
**30***	24.62	2984	Eicosamethylcyclodecasiloxane	N/A	767					73, 147, 221, 355, 281, 429, 207, 356, 295, **219**	3	
**31***	26.64	3228	Ergosterol	4	731					73, 69, 55, 75, 143, 129, 253, 81, 157, 169, **363**	2	[7]
**32***	26.83	3246	Stellasterol	N/A	917					**75**, 255, 69, 343, 55, 81, 73, 107, 105, 229	3	
**33****	2.718		Sucrose			$C_{12}H_{22}O_{11}$	342.1162	387.1191 $[M+HCOO]^-$	5.4	387 > 341	2	[8]
**34****	3.243		Unknown 1					290.0730		290 > 167	4	
**35****	4.789		3,12,20-Trihydroxy-7,11,15-trioxolanost-8,16,24-trien-26-oic acid			$C_{30}H_{40}O_8$	528.2723	527.2646	0.8	527 > 509, 465, 421, 317	2	[9]
**36****	5.154		Ganodernoid F			$C_{31}H_{44}O_8$	544.3036	543.2933	5.5	543 > 499, 481, 437, 367, 301	2	
**37****	5.519		Ganoderic acid I			$C_{30}H_{44}O_8$	532.3036	531.3033	13.2	531 > 513, 469, 401	1	[9]
**38****	5.774		Ganoderenic acid C			$C_{30}H_{44}O_7$	516.3087	515.3044	5.8	515 > 497, 481, 453, 435, 383, 193	1	[9]
**39****	5.964		Ganoderic acid C2			$C_{30}H_{46}O_7$	518.3244	517.3163	1.6	517 > 499, 481, 455, 437, 303, 287	1	[9–11]
**40****	6.741		3,12-Dihydroxy-4,4,14-trimethyl-7,11,15-trioxolanost-8,9,20,22-en-26-oic acid			$C_{30}H_{40}O_8$	528.2723	527.2673	4.4	527 > 525, 509, 483, 465		[10]
**41****	7.289		Ganoderic acid C6			$C_{30}H_{42}O_8$	530.2880	529.2809	0.4	529 > 527, 511, 467	1	[9–11]
**42****	8.131		Ganoderenic acid B			$C_{30}H_{42}O_7$	514.2931	513.2929	13.8	513>469, 436, 301, 265	2	[9–11]
**43****	8.185		Ganoderic acid B			$C_{30}H_{44}O_7$	516.3087	497.2941 $[M-H_2O-H]^-$	6.4	497>453, 438, 409, 303, 287, 249	1	[9–11]

Table S1. *Cont.*

**44****	8.189	Ganoderenic acid A	$C_{30}H_{42}O_7$	514.2930	513.2930	14.0	513 > 495, 479, 465, 451, 433, 403, 381, 363, 301, 193	2	[9]
**45****	8.244	Ganoderic acid K	$C_{32}H_{46}O_9$	574.3142	555.3039 $[M-H_2O-H]^-$	13.7	499 > 481, 451, 437, 421, 367	1	[9–11]
**46****	9.093	Ganoderenic acid K	$C_{32}H_{44}O_9$	572.2985	571.2916	0.5	553 > 538, 511, 494, 467, 449, 303	2	[9]
**47****	9.409	3,7-Dihydroxy-11,15,23-trioxolanost-8,16-dien-26-oic acid	$C_{30}H_{42}O_7$	514.2930	513.2860	0.4	513 > 495, 479, 433, 301, 193	2	[9]
**48****	9.734	12-Acetoxy-3-hydroxy-7,11,15,23-tetraoxolanost-8,20-dien-26-oic acid	$C_{32}H_{42}O_9$	570.2829	569.2869	19.8	513 > 495, 451, 433, 301, 193	2	[9]
**49****	9.775	Ganoderic acid H	$C_{32}H_{44}O_9$	572.2985	571.2887	4.5	571 > 553 > 511, 467	1	[9–11]
**50****	10.493	Ganoderic acid A	$C_{30}H_{44}O_7$	516.3087	515.2978	4.5	515 > 497, 435, 301	1	[9–11]
**51****	11.284	12-Hydroxy-3,7,11,15,23-pentaoxo-lanost-8-en-26-oic acid	$C_{30}H_{40}O_8$	528.2723	527.2687	20.1	527 > 509	2	[11]
**52****	11.670	12-Acetoxy-3-hydroxy-7,11,15-trioxolanost-8,16,24-trien-26-oic acid	$C_{32}H_{42}O_8$	554.2880	553.2745	11.2	553 > 511, 494, 467, 449, 301, 263	2	[9]
**53****	11.703	7,12-Dihydroxy-3,11,15,23-tetraoxo-lanost-8,20-dien-26-oic acid	$C_{30}H_{40}O_8$	528.2723	527.2721	13.5	527 > 509	3	[9]
**54****	12.148	Ganoderenic acid D	$C_{30}H_{40}O_7$	512.2774	511.2701	7.24	511 > 495, 478, 449, 434, 405, 285, 261	1	[9–11]
**55****	12.494	12-Acetoxy-7-hydroxy-3,11,15-trioxolanost-8,16,24- trien-26-oic acid	$C_{32}H_{42}O_8$	554.2880	553.2753	12.1	511 > 493, 449, 434	2	[9]
**56****	12.928	Ganolucidic acid A	$C_{30}H_{44}O_6$	500.3138	499.3065	5.2	499 > 482, 437, 419, 285, 269, 213	2	[9]
**57****	13.342	12-Acetoxy-7-hydroxy-3,11,15-trioxolanost-8,20- dien-26-oic acid	$C_{32}H_{42}O_9$	570.2829	569.2725	5.4	569 > 551 > 277	2	[9]
**58****	13.514	12-Acetoxy-3,7,11,15,23-pentaoxolanost-8,20-dien- 26-oic acid	$C_{32}H_{40}O_9$	568.2672	567.2600	16.7	549 > 507, 463	2	[9]

Table S1. *Cont.*

**59****	14.548	Ganoderenic acid F	$C_{30}H_{38}O_7$	510.2618	509.2656	21.8	509 > 491, 473, 465, 301, 261, 247, 217, 175	2	[9]
**60****	14.694	Unknown 2			533.3317		533 > 487, 469		
**61****	14.913	Unknown 3			535.3326		535 > 489, 371		
**62****	15.158	Unknown 4			535.3543		535 > 445, 419		
**63****	16.761	Unknown 5			553.2384		533 > 531, 487		
**64****	18.024	Unknown 6			483.2261		483 > 465, 439, 421, 287, 233		
**65****	18.415	Unknown 7			483.2673		483 > 465, 421		
**66****	19.336	Unknown 8			476.2464		476 > 279		
**67****	19.975	Unknown 9			341.1687		341 > 320, 297, 279, 159		
**68****	22.089	Unknown 10			295.2411		295 > 277, 195, 171, 113		
**69****	22.560	Unknown 11			293.1830		293 > 275, 249, 197, 185		
**70****	22.931	Unknown 12			527.3350		527 > 525, 485, 467		
**71****	23.007	Unknown 13			311.1089		311 > 182		
**72****	25.556	Unknown 14			475.2102		475 > 431		
**73****	25.801	Unknown 15			475.2904		475 > 431		
**74****	29.860	Unknown 16			355.2920		355 > 309		

[a]Metabolites were selected according to VIP > 1.0 and p-value < 0.05 in PLS-DA (*analyzed by GC/MS, **analyzed by LC/IT-TOF-MS); [b]Retention time (RT) were corrected by data pre-processing software; [c]Retention index (RI) metabolites analyzed by GC/MS were normalized with n-alkanes; [d]The level of metabolite identification according to Metabolomics Standards Inititative [12]; [e]The structure were deduced from previous reports

References

1. Isidorov, V.A.; Lech, P.; Żółciak, A.; Rusak, M.; Szczepaniak, L. Gas chromatographic–mass spectrometric investigation of metabolites from the needles and roots of pine seedlings at early stages of pathogenic fungi *Armillaria ostoyae* attack. *Trees* **2008**, *22*, 531.
2. Birkemeyer, C.; Kopka, J. Design of Metabolite Recovery by Variations of the Metabolite Profiling Protocol. In Proceedings of the Concepts in Plant Metabolomics; Nikolau, B.J., Wurtele, E.S., Eds.; Springer: Dortrecht, The Netherlands, 2007; pp. 45–69.

3. Erxleben, A.; Gessler, A.; Vervliet-Scheebaum, M.; Reski, R. Metabolite profiling of the moss *Physcomitrella patens* reveals evolutionary conservation of osmoprotective substances. *Plant Cell Rep.* **2012**, *31*, 427–436.
4. Isidorov, V.A.; Kotowska, U.; Vinogorova, V.T. GC Identification of Organic Compounds Based on Partition Coefficients of Their TMS Derivatives in a Hexane-Acetonitrile System and Retention Indices. *Anal. Sci.* **2005**, *21*, 1483–1489.
5. Kim, J.-S.; Chung, H.Y. GC-MS analysis of the volatile components in dried boxthorn (*Lycium chinensis*) fruit. *J. Korean Soc. Appl. Biol. Chem.* **2009**, *52*, 516–524.
6. Yaşar, A.; Üçüncü, O.; Güleç, C.; İnceer, H.; Ayaz, S.; Yayl, N. GC-MS Analysis of Chloroform Extracts in Flowers, Stems, and Roots of *Tripleurospermum callosum. Pharm. Biol.* **2005**, *43*, 108–112.
7. Radulović, N.S.; Đorđević, N.D. Steroids from Poison Hemlock (*Conium maculatum* L.): A GC-MS analysis. *J. Serbian Chem. Soc.* **2011**, *76*, 1471–1483.
8. Qian, Z.; Zhao, J.; Li, D.; Hu, D.; Li, S. Analysis of global components in *Ganoderma* using liquid chromatography system with multiple columns and detectors. *J. Sep. Sci.* **2012**, *35*, 2725–2734.
9. Cheng, C.-R.; Yang, M.; Wu, Z.-Y.; Wang, Y.; Zeng, F.; Wu, W.-Y.; Guan, S.-H.; Guo, D.-A. Fragmentation pathways of oxygenated tetracyclic triterpenoids and their application in the qualitative analysis of *Ganoderma lucidum* by multistage tandem mass spectrometry. *Rapid Commun. Mass Spectrom.* **2011**, *25*, 1323–1335.
10. Wu, L.; Liang, W.; Chen, W.; Li, S.; Cui, Y.; Qi, Q.; Zhang, L. Screening and Analysis of the Marker Components in *Ganoderma lucidum* by HPLC and HPLC-MSn with the Aid of Chemometrics. *Molecules* **2017**, *22*, 584.
11. Yang, M.; Wang, X.; Guan, S.; Xia, J.; Sun, J.; Guo, H.; Guo, D. Analysis of triterpenoids in *Ganoderma lucidum* using liquid chromatography coupled with electrospray ionization mass spectrometry. *J. Am. Soc. Mass Spectrom.* **2007**, *18*, 927–939.
12. Blaženović, I.; Kind, T.; Ji, J.; Fiehn, O. Software Tools and Approaches for Compound Identification of LC-MS/MS Data in Metabolomics. *Metabolites* **2018**, *8*, 31.
